# Miscellanies

**Published:** 1837-10-01

**Authors:** 


### MISCELLANIES.

### British Association.

The great and flourishing port of Liverpool had this year the honour and the pleasure of receiving the British Association during its seventh Anniversary —and we might say Jubilee. Nearly two thousand annual members entered between the 11th and 15th September—the largest assemblage, by at least 500, that has yet been collected of scientific and literary men, since the formation of the Institution. It is not for us to speak of any but the medical section; yet we may just observe, en passant, that the *intellectual* banquet at the different sections, from 11 till 3 o'clock, every day, was equalled, at least, by the sumptuous repast spread out at five o'clock, for the physical appetite.—Turtle soup, venison, game, and several wines, with various ceteras, disappeared as if by magic, from before 600 sçavans! It seemed as though the exercise of the intellect in the mornings had wonderfully improved the digestion in the afternoons; and that the labourers among trap, and gneiss, and granite, changed their occupations, with infinite delight, into mining among sirloins, turkies, grouse, and hams, at Lucas's rooms, from 5 till 7 o'clock. At 8 o'clock the Amphitheatre or the Town-hall became the theatres of reunion, where a multitude, composed of many thousands, male and female, assembled to hear lectures and speeches, in the former locality, and enjoy conversation and ices in the magnificent suite of rooms belonging to the corporation at the Town-hall.

The geological section always presented the greatest crowd of auditors. It is evident that old mother Earth—our parent and executioner—our cradle and sepulchre—attracts more attention, at this moment, than any other subject of investigation. The secrets of the prison-house are coming out, and the graves of countless myriads of generations are being opened to the view of man. In these, the last of created beings—man himself—sees the remains of animals and plants that existed millions of millions of years before he saw the light of sun or moon.

The medical section, during the first two days, was not so well attended as we expected. We hoped to have seen most of the distinguished practitioners of Liverpool, Manchester, and Birmingham there, especially as the rail-road has now annihilated space between those great towns; but we were much disappointed. Papers were sometimes read almost to empty benches, and it was only for an hour or two each day that any considerable number of medical men were collected together in their own section!

Dr. Williams opened the business by reading the report of the London committee appointed to investigate the cause of the sounds of the heart, in health and disease. The committee has come to the conclusion that the first sound is caused by the contraction of the ventricle itself—and the second by the reflux of blood against the semilunar valves. This is probably the truth. With respect to the abnormal sounds, the conclusion is that wherever we have the bruit de scie, bruit de soufflet, &c. there is some narrowing of the vessels or disease of the valves, offering obstruction, more or less, to the free transmission of blood from the heart along the arteries. Experiments were shewn in illustration, where jets or currents of water were thrown through gum-elastic tubes, and where pressure on the sides of the tubes produced (according to the degree) a close imitation of all the different bruits which we hear in diseases of the heart or great vessels. Although the principle was acknowledged, yet it was doubted whether considerable excitement of the circulation, mental or muscular, might not sometimes produce a bellows sound, which bruit disappeared when the circulation became tranquil. To this last opinion we ourselves subscribe, for reasons grounded on observation. Dr. Williams, however, would not admit that any excitement of the circulation could produce the bruit, unless some mechanical impediment was presented to the current of blood.

A very able paper on the physical appearances and chemical properties of pus was read by Mr. Brett, and excited much attention and approbation. For the particulars we refer to the account copied from the Athenæum, which presents a very fair account of the proceedings of this as well as of the other sections.

A demonstration or lecture by Dr. Macintosh, on the pathology and treatment of dysmennorrhœa, was listened to by a large audience, with excessive interest. As the non-professional journals could not, of course, give any detailed account of this demonstration, we shall be somewhat more particular than usual on this subject. Dr. M. laid on the table at least a score of uteri, all presenting morbid contraction or stricture of the os uteri, in a greater or less degree. Some of them were all but closed—some would scarcely admit a pig's bristle, and all were abnormally contracted. With the history of most of the cases he had been acquainted, and they all laboured under painful, and generally speaking, scanty menstruation. This contraction of a natural aperture he regarded as the result of some inflammatory action in the parts themselves or in their vicinity, more frequently than of any other cause. The straining of the uterus to get rid of the secreted menses, might explain all or most of the phenomena of dysmenorrhœa. The treatment, independently of constitutional means, consisted chiefly in removing inflammation, by the application of leeches (through the medium of a tube) to the os uteri itself—and where chronic contraction obtained, to introduce metallic bougies till proper dilatation was procured. He related several most important and interesting cases in illustration. The patients were chiefly dispensary patients; but afterwards he began to have private practice in the complaint, especially among the wives, daughters, and relations of medical men. It was objected that such operations would not be submitted to by English females. But Dr. M. triumphantly replied that he never *urged*—seldom indeed *proposed* the operation. He explained to the parties the nature of the disorder, and the remedy which he had sometimes found successful; but recommended the patients to try all other means, and, if they pleased, other practitioners, for the cure of their maladies. After a time, the patients themselves or their friends would propose the operation, which he always refused to do, till ardently solicited to do so. In this way he steered clear of all scandal.

Sir James Murray, of Dublin, exhibited his apparatus for the removal of atmospheric pressure from the whole or any part of the body—and also for the application of increased pressure to any particular portion of the body's surface. The demonstration was given with such humour and good nature—with so much of the "utile et dulce," that a large audience was delighted and instructed by the lecture. The author was greeted at the end with rounds of applause. But we shall now extract from the Athenæum a condensed account of the transactions of the medical section.

### SECTION E.—ANATOMY AND MEDICINE.

*President*—William Clark, M.D.

*Vice Presidents*—James Carson, M.D. F.R.S., Peter Mark Roget, M.D., Sec. R.S., Robert Bickersteth, Esq. Professor R. T. Evanson, M.D. M.R.I.A.

*Secretaries*—James Carson, jun. M.D. J. R. W. Voss, M.D.

*Committee*—Neil Arnott, M.D. F.R.S., Richard Bright, M.D. F.R.S., Hugh Carlise, M.D., James Copland, M.D. F.R.S., Professor Richard T. Evanson, M.D. M.R.I.A., Richard Formby, M.D., Augustus B. Granville, M.D. F.R.S., John Houston, M.D. M.R.I.A., James Johnson, M.D., James Macartney, M.D. F.R.S., Charles Herbert Orpen, M.D., William Henry Porter, Esq., Charles B. Williams, M.D. F.R.S., John Yelloly, M.D. F.R.S.

Dr. Roget stated that he presided on this occasion, owing to the absence of

Professor Clark, whose arrival was expected.

The second report of the Sub-Committee, appointed by the Association to investigate the Motions and Sounds of the Heart, was read by Dr. Charles Williams.

Before describing their last investigations, the Committee stated that they had found frequent opportunities of confirming the conclusions of their former researches on the natural sounds of the heart; and these conclusions not having been shaken by any subsequent experiment, or well-founded objection, the Committee consider them established; viz., that the first sound of the heart is *essentially* caused by the sudden and forcible tightening of the muscular fibres of the ventricles when they contract; and that the second sound essentially depends on the reaction of the arterial columns of blood on the semilunar valves of the arterial orifices, at the moment of the ventricular diastole. Certain other circumstances were stated, as being capable of adding to, or modifying these sounds.

The chief subjects of their present inquiry were, the unnatural, or morbid sounds, sometimes heard in the heart and arteries; and in investigating the causes of these sounds, which Laennec compared to blowing, filing, sawing, purring and cooing, or musical sounds, they sought to determine, 1st, What is the essential physical cause of these sounds; and 2nd, In what manner disease can develope this physical cause —a correct answer to these inquiries would determine the value of these sounds as signs of disease.

The Committee found that they could produce precisely the same sounds in every variety, by impelling, in various modes and degrees, a current of water through India-rubber tubes; and by numerous experiments, they ascertained the relations which the character of these sounds bore to the nature of the impediment, and to the force of the current. They obtained similar results on experimenting on the arteries of living animals; and discovered, that in the human subject the same sounds may be produced by simple pressure, not only in the arteries, but in the veins also. They found that the sounds heard in the neck, described by some eminent French writers under the names, "bruit de diable" and "bruit de mouche," as signs of a particular morbid condition, which requires the use of certain remedies, may be produced at will, by the pressure of the stethoscope on the jugular veins of the healthiest persons, and is therefore not necessarily a sign of disease, but has probably been accidentally caused by the same pressure in many cases in which it has been considered as a morbid sign.

The Committee conclude, in answer to the first inquiry, that a certain resistance to a moving current is the essential physical cause of all the various sounds in question, and that this resistance is generally given by some pressure on, or impediment in, the tube through which the current moves; but that sometimes the resistance is caused by a change in the direction of the current, by which it is made to impinge on the walls of the vessel which contains it.

The second inquiry the Committee think can be fully answered only by extensive clinical and pathological observation, with due regard to the previous investigations; but they have planned some experiments, that promise to elucidate certain obscure points of the pathology and diagnosis of diseases of the heart and arteries, the knowledge of which would be of direct practical advantage. These points the Committee propose to investigate, if the Association think fit to re-appoint them to this office.

The thanks of the Section were voted to the Sub-Committee, and the members were requested to continue their labours.

Dr. Copland said, the Report stated that the heart beat after the cessation of respiration, which he believed, for the heart was the *ultima moriens.*—Dr. Carlisle concurred with the Report in its general view as to the cause of the sounds of the circulation in tubes from obstruction to the course of the fluid current, but differed from them on the explanation of the systolic sound, which, in his opinion, was owing to resistance from the irregularities of the heart's surface. The heart, in these cases, was *ultima moriens*, and would act, though it contained no blood, contrary to Bichat's opinion.—Dr. Granville could confirm, from his experiments with poison, chiefly prussic acid, the solitary instance adduced by Dr. Williams, of the beating of the heart on the cessation of respiration, and that it continued its action, though without blood. He found, and five other gentlemen were present timing with their watches, that in seven experiments on animals made in London, the fibres of the heart were alive after the death of the lungs (i. e. if life could be pronounced from dilatation and contraction)—in one case five minutes, in a second eight minutes, in a third eleven minutes; and on touching with a scalpel to a much longer period; and in this case not a drop of blood was contained in the heart. The action was not owing to blood or fluid, but was merely mechanical; and it was found, in dogs and cats destroyed by prussic acid, that, on opening the cavity of the chest and abdomen simultaneously, respiration had ceased, the lungs were collapsed, but the heart beat, and the intestines acted peristaltically. The heart, as a muscle, does most certainly survive respiration, and is the *ultima moriens*.

Dr. Johnson said the Report concluded that all abnormal sounds were indicative of disease or morbid obstruction; from which he must dissent. He believed the excess of the velocity of the circulating fluid was sufficient to account for it, without the supposition of any obstruction from morbid contractions, and other impediments in the arterial tubes. He was, himself, an instance of the truth of this; for when his circulation was increased, either by excitement, food, or running, he had heard these sounds in himself, and he had equally verified it by observations on others; he could not, therefore, consider these sounds as certainly abnormal. He would ask Dr. Williams, if a trial had been made of the effect of velocity of fluids through tubes with the view of ascertaining sounds?—he would ask, if sound was produced at the extremity of the vessel, why is it not heard in the diastole of it, the blood issuing, as some say, in jets, or from reflux?—Dr. Williams stated, in answer, first, that the Committee had examined this subject physically, when they found some contraction, or obstruction, or bend, was necessary for the production of the murmur and other sounds; but that they had not entirely sifted the physiological part of the subject, though he believed that their physical labours were fully applicable to it. To have sounds in currents, there must be some resistance, as friction. In the natural condition of the vessel, this increased velocity of Dr. Johnson's might create friction, from want of dilatability causing the murmuring sounds; and he would certainly recommend Dr. Johnson and his friends not to repeat their experiments frequently—at least, on themselves. To Dr. Johnson's second question—why is there not sound in a continuous current?—it would depend on the pressure; according to its degree, so would be the sound—if considerable, the *bruit de diable*. He now wished to refer to the objections of Dr. Carlisle. In last year's Report, they attributed the essential sounds of the heart to the muscular action of the heart, not to the blood; for they found the sounds perceptible, but in a diminished degree, without blood. In answer to the question, why the sounds of the heart should differ from those of the artery, he should say, the motion of the heart was only commencing, had not yet acquired momentum, and the blood was expanded over a large surface—that the ventricle contracted towards a centre, which was the artery—and that there was very little friction on the internal surface of the heart, though he could not tell how much to attribute to the *Carneæ columnæ* alluded to by Dr. Carlisle; but if examined, they would see very little irregularity where the blood converged, the principle being towards the bottom. The blood ran with a different momentum in the arteries than it did in the heart, being only about three inches in half a second, and the slightest obstruction must, therefore, oppose it, while in the heart the blood moved as the muscle of the heart contracted, and other obstructions moved. As to the character of the sounds, they resembled those of natural objects, and the natural differed from the morbid. The natural was a dumb percussive sort of sound, the morbid was whizzing, giving an idea of friction—filing, sawing, rushing, and so on, which an accustomed ear could distinguish with precision.—Dr. Williams replied to a question asked by Dr. Granville, that the general result of their investigations led to the conclusion that velocities of motion did not alone produce sound; there must be some obstruction, some resistance.

Mr. Brett then read a paper 'On the Physical and Chemical characters of Expectoration in different Diseases of the Lungs, with some Preliminary Remarks on the Albuminous Principles existing in the Blood.'

The remarks on the blood referred more particularly to a general view of the albuminous principles existing in that fluid. The simplest view which could be taken of the vital fluid, is that which refers its constitution to a mixture of fluid, or soluble, and insoluble albumen, —the one constituting what is termed the serum, the other the crassamentum, or cruor. The author of the paper then proceeded to relate the different opinions which had been published on the specific gravity of the blood, quoting the statements of Berzelius, Gmelin, Dumas, and other chemical philosophers of distinction; at the same time remarking, that all these statements did not differ materially from each other, and might be considered as depending upon the fact, that the specific gravity of the blood might differ slightly, not only in different individuals, but in the same individual at different times. He then noticed the different modifications of albumen existing in the serum, which he divided into three forms:—1st, Soluble or free albumen, capable of undergoing coagulation by heat; 2ndly, Albumen in combination with a basic body, viz. soda; and, 3rdly, A form of albuminous matter, which he termed the colourless self-coagulating albuminous principle.

The crassamentum, as it is commonly called, of the blood, he also considered as made up of more than one form of *solid albuminous* matter; viz. of solid albumen capable of undergoing decoloration by ablution with water, and of solid albumen incapable of being decolorated by the same process; the former being insoluble, and constituting what is commonly understood under the name of fibrine, the latter soluble in water, and frequently designated red particles or *hæmatosine*. Some remarks then followed on the microscopic examination of the blood, and on the different forms of the globules in different animals. The author then proceeded to detail the various physical characters of the expectoration in the healthy condition of the lungs, as well as in its varied morbid states. The physical characters of saliva were entered upon, and the globularity of its opaque portions alluded to. The physical characters of expectoration in the pituitous catarrh of Laennec were then detailed; also those of the expectoration in acute and chronic bronchitis—in hæmoptysis, or pulmonary apoplexy—in pneumonia—and lastly, in different stages of phthisis. The chemical characters of these different modifications of expectoration were then fully treated of, and reference made to a tabular arrangement which the author had embodied in his papers, exhibiting the action of certain re-agents —first, on saliva, and then on the different forms of sputa, the physical characters of which had been already fully noticed. It was remarked, that saliva did not contain any soluble albumen capable of undergoing coagulation by heat; neither did it contain any solid albuminous matter, the main bulk of the solid contents of that secretion being mucous. The mode of analysis adopted, was, to deject saliva in cold water, and then subject the filtered fluid to the action of certain re-agents; another portion of saliva was then dissolved in a caustic alkali, and the alkaline solution subjected to the agency of certain tests. The quantity of solid matter in a given weight of saliva was also announced, as well as the saline matters, and their chemical nature stated: different authorities bearing on the subject were quoted, especially the statements of Berzelius and L. Gmelin. The chemical characters of expectoration in pituitous catarrh were then described, and a mode of analysis was stated to have been adopted, analogous to that employed in the case of saliva: this modification of sputum was regarded as purely mucus, possessing no albuminous matter; it was found to contain a very small proportion of solid matter in a given weight, but the quantity of saline matter was found to be considerable, when compared with the quantity of solid matter; and this saline matter the author's experiments lead him to conclude was diminished in quantity as the disease progressed. The chemical nature of sputum of the acute and chronic bronchitic character was then entered upon, and noticed as differing in certain respects from the preceding form of expectorated matter, in containing, for example, a much larger proportion of solid matter in a given weight than was found in simple pituitous expectoration, and generally a smaller proportion of saline matter; it also differed in containing, generally speaking, small quantities of soluble albumen capable of undergoing coagulation by heat.

Pneumonic expectoration was then treated of, and noticed as principally made up of a tough mucoid secretion intermixed with blood, to which last was owing its peculiar rust or brick-red colour, and also its powers of undergoing, to a certain extent, coagulation by heat when mixed with water and filtered; it was also found to differ from most other forms of expectorated matter, in containing no inconsiderable quantity of oxide of iron, derivable from the blood with which it is impregnated. Phthisical expectoration was the last form of sputum, the chemical characters of which were described. It was noticed as differing materially in different stages of the disease; in the earlier and middle stages scarcely not at all; for the most part, at least, differing from the expectoration met with in chronic or acute bronchitic affections: in the latter stages, however, not unfrequently possessing the characters of a simple collection of puriform matter, containing very large quantities both of soluble and solid or insoluble albumen, much solid matter also in a given weight, with the ordinary saline matters found in other varieties of sputa, superadded to which was a notable proportion of oxide of iron. It was stated, that in no disease, except phthisis, did the expectoration contain so much soluble albumen capable of undergoing coagulation by heat; and also in no disease, except pneumonia, was there so large a proportion of solid matter in a given weight of the expectoration: this observation referring, however, to the sputum in the latter stages of phthisis, where it puts on the character of a collection of puriform matter. Allusion was then made to the fatty matter existing in expectorated fluids, which was found to be the same in quality in almost every variety of sputum, but differing in quantity, being much greater in quantity in well-marked phthisical expectoration than in any other variety. The fatty matter was peculiar, too, from the high temperature which it required for its fusion, it being considerably higher than that necessary for the fusion of the more ordinary forms of fatty matter, and even higher than that required for cholesterine: this fatty matter was soluble in alcohol and ether, being deposited from the former when its boiling solution cooled. The author also referred to the power which a galvanic current, even of low intensity, possessed of coagulating the aqueous fluid, obtained either by digesting saliva, or any of the modifications of expectorated matter before alluded to, in water, and filtering the fluid. This coagulation was not regarded by the author as proving the presence of albumen, because, in cases in which the galvanic current effected the change in question, the most delicate re-agents with which chemists are acquainted for the detection of albumen, failed to detect the slightest trace. The author then detailed his experiments on crude and softened tuberculous matter; he submitted the former to the action of the same re-agents as he employed to re-act upon ordinary fibrine, and was led to conclude that the crude tubercle did not differ chemically from solid albumen or fibrinous matters. The mode of analysis employed in examining the crude and softened tubercle was the following—it may be observed, that the crude tubercle was examined side by side with ordinary fibrine: the crude tubercle was dissolved in a weak solution of caustic potass; a similar solution of fibrinous matter was obtained, and both submitted to the action of the same re-agents, with results as nearly similar as possible. The agents employed were the mineral acids, acetic acid, and ferrocyanide of potassium, tinct. galls, corrosive sublimate, &c. The softened tuberculous matter was first dejected in water, and then filtered; the filtered fluid, when submitted to re-agents, was found to contain soluble albumen; that portion of the tubercle insoluble in water, was dejected in a weak alkaline fluid, by which a solution was obtained. This alkaline solution, when submitted to the necessary re-agents, indicated the existence of solid albuminous matter or fibrine; hence, the softened tubercle was regarded as analogous in its chemical characters to purulent matter. Experiments were then made on the tuberculous matter which had undergone perfect softening, and the result was, that the latter was chemically identical with pus; from which it was deduced, that fibrinous matter was, by a process of softening or fluifaction, converted into actual pus, and hence a fruitful source of the abundantly albuminous fluid found in the expectoration of patients in the latter stage of phthisical disease. The author then concluded his paper, by stating the results of a quantitative analysis of the expectoration of a marked puriform character, obtained from a patient in the last stage of phthisis. It was found to consist of—water; albuminous matter, with a little mucus; extractives, soluble in alcohol; ditto, soluble in water; fatty matter; saline matters, consisting of the alkaline chlorides, phosphates and carbonates, with earthy phosphates and oxide of iron.

Dr. Williams expressed his gratification that the labours of so expert a chemist had been directed to so important a branch of science as animal chemistry, which had been strangely neglected, and almost overlooked. This he lamented the more, since the subject would promote both the theory and practice of medicine. He would, however, ask Mr. Brett if he had observed in his microscopical experiments in early phthisis, what the French had detected in the sputa, a sort of downy substance, peculiar to the very early stages of this disease, and only observable through the microscope; indeed it was a microscopic phenomenon. It was a novelty to him, that tubercles by softening, as mentioned by Mr. Brett, assumed globular forms of which they were devoid at the commencement, a fact confirmed by Gmelin. This paper pleased him, for it further assured him of the truth he had taught, that softening was a modification of suppuration, a conversion of albumen into pus. There was a total absence of organization in tubercle. Mr. Brett had not observed the microscopical flocculent and woolly appearance, for his attention had not been so directed. Dr. Williams explained, that it did not resemble flocculi, but was, he considered, a microscopical appearance only. Mr. Brett had made his microscopical observations on what was obvious to the naked eye, and opaque portions of these secretions he found to be globular, not so perfect in cheesy suppurations as in those of other characters. Mr. Williams enquired what was the opaque compound in saliva. Mr. Brett answered, it might be a modification of albumen, but he could not say exactly. Dr. Roget remarked, that the paper made it appear that the formation of globular inexpectorated matter was spontaneous. Dr. Williams stated, that the belief of this sort of globular formation might be supported by the circumstance of its production in serum, after it had been some time at rest, which had been shewn by Dumas and Sir Everard Home. Mr. Brett did not consider the cases parallel, since serum was highly albuminous, and this expectoration not. Dr. Johnson thought, that, independently of the chemical acumen, the paper would be of value to practitioners, on account of the physical character of expectorated matter, of which there were three modifications: 1st, Mucus, from intensity of disease in the bronchia; 2nd, Tuberculous matter softened down, and escaping into the bronchial tubes, or entirely discharging from them; 3rd, Secretion of purulent matter from excavated surfaces, scarcely distinguishable from the matter of abscess, or the after discharge from the depôt, by which he meant the matter secreted after the first contents of an abscess had been discharged. Mr. Golding Bird, after alluding to Mr. Brett's experiments, and drawing the attention of the Section to those parts which particularly merited the attention of practitioners, offered a suggestion, in explanation of the circumstances mentioned by Mr. Brett, in which mucus, previously limpid, became opaque, and deposited globular masses of albumen, after a few hours' exposure to the air; as well as of the fact alluded to by Dr. Williams, viz. the circumstance of serum of blood becoming opaque, and depositing globules by exposure. The rationale of these two facts Mr. Bird conceived might depend upon the action of carbonic acid upon albumen, and upon alkaline albuminates, first pointed out by him in a series of papers published in the Philosophical Magazine. In the case of mucus, he believed that a notable portion of albumen was present, held in solution by carbonic acid, and that, by exposure to air, or to an atmosphere of hydrogen, this solvent was abstracted in an exceedingly gradual manner, in consequence of which, albumen was deposited in the form of rounded particles. In the case of serum, the converse Mr. Bird believed to take place; for, as Mr. Brett has shewn in his paper, this fluid to contain, independent of free albumen, a small portion of albuminate of soda, it appeared reasonable to suppose that the carbonic acid present in the atmosphere acted on, and decomposed this compound, forming carbonate of soda, and causing the deposition of albumen in a globular form. This hypothesis, moreover, serves to explain the fact of serum, previously neutral, becoming alkaline after a few hours' exposure, for the albuminate of soda previously existing, is without action on test papers, whilst the carbonate of soda, produced in the manner above mentioned, is fully capable of producing an alkaline effect on turmeric paper. This explanation is in direct accordance with the experiments related in the Philosophical Magazine, before alluded to.

Dr. Carson in the chair.—Dr. Holland read a paper "On the cause of Death from a blow on the Stomach, with remarks on the means best calculated to restore animation suspended by such accident."

The writer commenced by stating, that the occurrence of death from a blow on the stomach has never received any full or satisfactory consideration. It is cursorily alluded to in treatises in which cases of sudden death, from a supposed impression made upon the nervous system, are discussed; and it is mentioned in this connexion, from being imagined to depend on the same mysterious cause. The cause of death from a blow on the stomach is referred to a shock communicated to the nervous system, by which the action of this organ is arrested. The primary impression is considered, by some, to be made upon the semi-lunar ganglion. The situation which this occupies, and not any peculiarly intimate connexion which it has with the heart, has suggested this explanation. Were it unequivocally shewn that the heart derives its contractile power from this ganglion, and that this is injured, or in any way affected by the blow, cause and effect would be too indissolubly united to admit of dispute. No one has, however, shewn that the heart receives its nervous energy from such source, nor are there any facts demonstrating that this ganglion is injured, or its functions disordered by a blow. No distinct evidence is, indeed, presented, proving that this occurs; writers on this subject speak only of suspended or deranged nervous action, and the effects of a shock on the nervous system.

The circumstances which have led to the adoption of the prevailing notion may, perhaps, be reduced to three :—1. Situation of the ganglion. 2. The spot where the blow is received. 3. And the consequent fatal effect. These circumstances are the only reasons adduced; and yet, without other corroborating facts, they are scarcely deserving of notice. If the plexus or semilunar ganglion be considered as a centre of nervous energy, this does not supply the heart or chest generally, but, indeed, the aorta and abdominal viscera: and hence a blow on the pit of the stomach, were its effects transmitted directly through the nervous system to the organ supplied by it, would be more likely to disturb the functions of these viscera than the action of the heart. It is not unphilosophical to contend, that an injury inflicted on this centre will disturb the organs dependent upon it; but the heart receives nervous influence from the brain, spinal cord, and sympathetic nerve, previous to the formation of the lunar plexus, or ganglion; and, therefore, if affected through either, it cannot be explained to depend on the deprivation of nervous fluid, but on the transmission of an impression or undulation.

In entering upon this inquiry, the first step was to determine the important organs peculiarly liable, from their situation and functions, to be deranged by a blow on the stomach. Those were the aorta and vena cava ascendens. The pit of the stomach is unquestionably the situation where these large and important vessels are alone liable to severe functional derangement from a blow. Above this point they are securely protected by the parietes of the chest, and below it by the mass of abdominal viscera. A blow in this situation has necessarily a tendency, whether it strike the artery or the vein, to urge the circulating fluid towards the heart. Nature, by means of the semilunar valves, has prevented the frequent occurrence of such an accident; but the violence of the blow is quite sufficient to overcome this obstacle or barrier to the retrograde motion of the blood. The fatal result is to be referred to the sudden propulsion of arterial fluid into the left ventricle, and not to the greater force with which the venous blood may possibly be returned to its destination. In discussing this subject, there are three points to which especial attention should be given. 1st. Is the aorta so situated as likely to be influenced in the manner stated? 2ndly. Would a blow, given with great violence, cause a retrograde motion of blood, and its entrance into the left ventricle? 3rdly. Would the latter circumstance be sufficient to cause death?

The latter part of the paper was occupied in endeavouring to establish the principles laid down; shewing, that death from a blow on the stomach is not, as has always been considered, referable to any injury or impression made on the nervous system.

Dr. Copland required an explanation of three questions. Had Dr. Holland observed the morbid appearances after death from blows of the stomach? 2ndly, Is it possible to produce the retrograde motion of the blood from the part of the artery impinged to the heart without rupturing the semilunar valves? 3rd, Had Dr. Holland much experience of the treatment?

Dr. Holland knew that his views admitted of discussion and of objection, since they differed so widely from the received notions. He had only observed one case, and it was fatal. The post mortem examination shewed a florid appearance of the mucous membrane of the stomach, especially at the pit, not unlike what is observed during digestion. The blood was fluid. With regard to the rupture of the valves, he could present no facts; but, in his opinion, the valves would not be seriously injured, and failure in resuscitation could not be attributable to the state of the valves.—Dr. Copland had not himself witnessed the morbid appearances of sudden death from a blow on the stomach, but several such were on record, which were sufficient to shew the influence of the ganglionic nerves. He thought retrogradation of the blood's current, as mentioned by Dr. Holland, could not take place without a rupture of the valves, or even of the heart itself. The heart could be injured in these cases physically, from the diaphragm as well as the vessels; but, in considering this kind of death, we are not to attribute its cause to any part of the body singly, but to take a comprehensive view, and to take in the whole collectively — the respiration — circulation—epigastrium—ciliary plexuses of nerves—though one might be more injured than another.—Dr. Johnson had had his attention directed to this subject from an early age. At school there was a boy a great pugilist, who, in his rencontres, gave what he called his heart-ing, meaning a blow over the region of the heart. The first, and principal effects, were a sudden suspension of respiration, which was relieved and followed by gasping; the heart, oppressed, did not cease its action, for, if it did, deliquium animi would ensue: the pain was very peculiar. He agreed with Dr. Copland as to the cause of death in these cases, and could not conceive that a forcible impression of the aorta could throw the blood back, and force the valves; for in dead bodies this was difficult, though the valves then perhaps were more flaccid; and here it was said to occur to the living—being still more difficult. —Sir James Murray had seen two cases of suspended animation from blows on the stomach; one recovered, and the other died. The remedy he should recommend, would be to throw a bucket of cold water over the body—gasping would ensue, and respiration follow. He could not agree with Dr. Holland, more especially when he considered the slight influence the small quantity of forced blood would exert. A space of the artery, equal at most to about two inches, would be struck—the blood contained in one inch would be distributed downwards through the iliac arteries to the extremities, where space enough was found; and the other inch of blood sent upwards, would find a much greater space through all the vessels of the chest and upper extremities. He would not deny that an inch of blood above the healthy quantity suddenly propelled into the heart might injure that organ, but thought there was not enough to injure the heart and vessels, as they must be, from sudden violent blows on the stomach.—Dr. Williams had not read any author who gave a satisfactory account of the effects of blows on the stomach, but he agreed in opinion with Dr. Copland. He had paid particular attention to the semilunar valves, and must differ from Dr. Johnson. They are perfectly mechanical, and act in the dead as in the living bodies. They completely stop all entrance to the heart, and must be ruptured for entrance there. Dr. Williams then alluded to death from syncope, by poisons, crushing of the limb, ingestion of cold water,—and concluded by recommending treatment by diffusible stimuli.

Dr. John Reid then gave an account of an experimental investigation into the Glosso-pharyngeal, Pneumogastric, and Spinal accessory Nerves. This communication was stated by Dr. Reid to contain merely a short epitome of some lengthened remarks, which he had drawn up on this subject; but it embraced the principal results, deduced from the numerous experiments which he had performed upon those complicated and important nerves, generally included under the eighth pair.

*Glosso-pharyngeal Nerve.*—The experiments on this nerve were all performed on dogs, and were 27 in number. Seventeen of these were for the purpose of ascertaining if it was to be considered a nerve both of sensation and motion, and what were the effects of its section upon the associated movements of deglutition, and on the sense of taste. The other ten were performed on animals immediately after they had been deprived of sensation, with the view of ascertaining more accurately how far it is to be considered a motor nerve. The phænomena observed in these experiments were first stated; and the conclusions drawn from a review of the whole of the data thus obtained were these:—first, that this is a nerve of common sensation; second, that mechanical and chemical irritation of this nerve before it has given off its pharyngeal branches, or of any of those branches individually, is followed by extensive muscular movements of the throat and lower part of the face; third, that the muscular movements thus excited depend, not upon any influence extending along the branches of the nerve to the muscles moved, but upon a reflex action, transmitted through the central organs of the nervous system; fourth, that these pharyngeal branches of the glosso-pharyngeal nerve possess endowments connected with the peculiar sensations of the mucous membrane, upon which they are distributed, though he cannot pretend to say positively in what these consist; fifth, that this cannot be the sole nerve upon which all these sensations depend, since the perfect division of the trunk on both sides, with removal of a considerable part of it (if care be taken to exclude the pharyngeal branch of the par vagum, which lies in close contact with it), does not interfere with the perfect performance of the function of deglution; sixth, that mechanical or chemical irritation of the nerve, immediately after an animal has been killed, is not followed by any muscular movements, provided care be taken to insulate it from the pharyngeal branch of the par vagum; seventh, that the sense of taste is sufficiently acute after the perfect section of the nerve on both sides, to enable the animal readily to recognize bitter substances; eighth, that it may probably participate with other nerves in the performance of the function of the sense of taste, but it certainly is not the special nerve of that sense; ninth, that the sense of thirst does not depend entirely upon this nerve.

*Pneumogastric, or Par Vagum Nerve.*—From the result of 30 experiments on this nerve, he is satisfied that severe indications of suffering are generally induced by pinching, cutting, or even stretching this nerve. Powerful respiratory movements were excited in some of the animals, when the trunk of the nerve was compressed for a few moments by the forceps.

*Pharyngeal Branches of the Par Vagum.*—From 17 experiments performed on dogs, either when alive, or immediately after being deprived of sensation, he concludes, that these are the sole motor nerves of the constrictors of the pharynx, the stylo-pharyngeal and palatine muscles; and that the sensitive filaments contained in these branches of the par vagum are exceedingly few, if under ordinary circumstances they exist at all.

*Pharyngeal Branches of the Par Vagum.*—From his experiments on these nerves, repeated and confirmed in various ways, he concludes, that all the muscles which move the arytenoid cartilages, receive their motor filaments from the inferior laryngeal or recurrent nerves. That one only of the intrinsic muscles of the larynx receives its motor filaments from the superior laryngeal, viz. the crico-thyroid muscle, and that consequently, the only change which this nerve can produce upon the larynx as a motor neive is, that of approximating the cricoid cartilage to the thyroid,—in other words, of shortening the larynx; and that the sensations of the larynx depend upon the superior laryngeal nerve. These experiments are completely subversive of the statement of Majendie, that the inferior laryngeal nerves supply those muscles only which open the glottis, while the superior supply those muscles which shorten the glottis. They also illustrate in a very satisfactory manner the causes of the dyspnœa, amounting in some cases to strangulation, when the inferior laryngeal nerves are cut or compressed.

He has also satisfied himself, that when any irritation is applied to the mucous membrane of the larynx in the natural state, this does not excite the contractions of these muscles, by acting directly upon them through the mucous membrane, but that this contraction takes place by a reflex action, in the performance of which the superior laryngeal nerve is the sensitive, and the inferior laryngeal is the motor nerve. He is also convinced that the muscular contractions of the œsophagus are not called into action by the ingesta, acting directly as an excitant upon the muscular fibre, through the mucous membrane, but by a reflex action, part of the œsophageal filaments acting as sensitive, and others as motor nerves. Our space will not permit us to state any of the results obtained from the experiments on the other branches of the par vagum.

*Spinal Accessory.*—This nerve was cut across in seven dogs at its exit from the cranium, and no effect upon the voluntary movements of the muscles of the neck could be observed. In other animals the nerve was first cut across on one side, and then a weak dose of prussic acid given. This was frequently followed by powerful, slow and regular respiratory movements, during some of which distinct contractions of the sternomastoid muscle were observed in unison with the other muscles of inspiration.

The next paper was entitled "Observations on the Structure of the Sacrum in Man and some of the lower classes of Animals," by Hugh Carlisle, M. B. Mr. Carlisle exhibited to the Section several anatomical preparations of the human sacrum in different states of growth, in which the separate formation of the lateral parts, consisting both of ribs and of transverse processes, was distinctly shewn. The analogous structure in certain classes of reptiles were displayed by means of preparations and original drawings, and the errors of descriptive anatomists on these subjects were pointed out. Mr. Carlisle shewed that some of the saurian reptiles afford the best examples of distinct and well-developed sacral ribs, although this peculiarity in their structure has been wholly overlooked by previous anatomists. In these animals the sacral ribs are two in number on each side, the anterior being articulated to the bodies of the last dorsal and the first sacral vertebræ, and to the intervertebral fibro-cartilage, the posterior to the last sacral and the first caudal vertebræ. In the human subject the sacral ribs are four on each side, and they remain in a separate and distinguishable state until the age of from three to seven years, after which period they are all, except in rare instances, consolidated with each other and with the bodies and transverse processes of the sacral vertebræ. The os ilium in the fœtal state, and for some years after birth, in the human subject, is connected to only the first two at each side of the sacral ribs, a fact which is consistent with the imperfect development, at that period, of the lower extremities, and with the disposition, at an early age, to walk on all fours, and which affords an additional example to those already known of the similarity which prevails between the temporary forms of certain parts of the human body and the permanent dispositions of the corresponding parts in animals of a lower class. In many of the quadrumana, of quadrupeds, and of reptiles, two is the number of sacral ribs constantly in apposition with the os ilium : in the human subject, at a more advanced period of growth, the os ilium at each side is connected by a cartilaginous intermedium to the extremities of three sacral ribs, and in one instance, in the skeleton of a negro, Mr. Carlisle observed it conjoined to four. This communication was concluded by some observations on the peculiarities of structure exhibited in the skeletons of the Testudo Græca and the Testudo Midas, the former of which Mr. Carlisle considers to possess *two* sacra, one for the posterior and one for the anterior extremities; while the latter has but one sacrum belonging to the posterior extremities, and, possessing a more extensive range of motion in the anterior, presents in them nearly the same mode of junction to the spine which prevails in birds and some quadrupeds.

A paper was then read by Dr. Black, on the Epidemic Influenza, as it occurred at Bolton-le-Moors, in January, February, and March of this year, and which principally referred to the meteorology of the season, and to the question how far the epidemic bore upon vital statistics and mortality.

After a summary of the principal pathognomonic symptoms which characterize the disease in its more severe and fatal forms, with a short notice of *post-mortem* investigations, and the treatment generally adopted, Dr. Black proceeded to say, that to the medical philosopher, the extent and intensity with which the epidemic bore upon the population of the country, along with the ratio of mortality which marked its progress, as well as the meteorological state of the weather, which preceded and accompanied its march over the kingdom, were subjects of great, and of historical interest, especially when compared with the nature and progress of former epidemics of the kind, and with the rise, abode, and intensity, in other places, of the same epidemic. For the purpose of elucidating these important and relative matters under which the disease appeared and prevailed at Bolton, he obtained a correct register of the weather in its principal meteorological conditions, for the months of January, February, and March of this year, during which the epidemic appeared, prevailed, and decayed at that place, to which he had added a column exhibiting a scale of the epidemic's rise, maximum intensity, and decay. This column was constructed from the several lists of cases of influenza entered and kept by three of the principal practitioners of the place and himself; which entries for each day being added together, gave a ratio corresponding to 100 as the maximum intensity on the 3rd February. To this table he also added a Mortality Register of 420 burials, and therein stated the several ages and quinquennial periods, at which the individuals died, after the fifth year, with the different amounts and ratios for the late epidemic season as well as for the average of the same months during the five previous years.

[We have not space for the tables, but the following inferences from them will be sufficient for our readers.]

From the register of the weather it is seen, that during the first two weeks of January, the temperature was very irregular, varying in the mean of morning, noon, and night from 47°.3 to 27°.3, while the barometer was gradually falling from 30.27 to 29.17, and snow, hail, rain, and fair weather prevailed by turns. The epidemic, during this period, had scarcely made its appearance, and except it had subsequently done so, the few cases of suffocative catarrh and atonic bronchitis would have been attributed to an endemic and sporadic origin. With the 14th day of the month commenced a week of fair weather, and of a steadier and milder temperature; but after a very sudden rise, a declining state of the mercurial column took place, which reached its lowest depression of 28.88 on the evening of the 21st, while the dew point became nearer to the mean temperature. Contemporaneous with this lowest state of the atmospheric pressure, on the 21st commenced the full and rapid invasion of the epidemic, similar to some mighty mortific wave, that was sweeping over the country, sudden in its attack, but more lingering on its departure. As the disease advanced, the temperature fell for seven days, with continued rain, or snow till the end of the month. The barometer on the whole gradually rose, until it attained 30.10 on the morning of the 4th of February. The previous day the epidemic had reached its maximum intensity, having, in the course of a fortnight, laid the whole population, with trifling exceptions, under its influence, which extended from the merest *malaise*, or slight catarrh, to the most deadly effect on the functions and organs of life. After this culminating point, it gradually diminished in the number of cases, though not in the severity of many individual instances; and many diseases occurred, even until March, which could be fairly attributed to the constitutional taint or diathesis, which the epidemic had produced. The invasion of the epidemic was preceded by and attended with easterly and southerly winds, while the atmosphere was much loaded with moisture. This high point of saturation may be frequently observed during the prevalence of the epidemic, for the dew point is, for several days, seen to be as high, if not higher, than the mean temperature of the day. This anomaly in part arises from the dew point being taken only once a day, at noon, while the temperature was not only taken at that hour, but at morning and night. The average of the three would therefore be sometimes below the dew point at noon.

In comparing the *mortality* of the five previous years, during the same months with that during the prevalence of the epidemic, it appeared that the increase on the whole three months was equal to 45 per cent. on the average mortality, and for the month of February alone, it was 160 per cent. Of the 420 deaths, 205 were males and 215 females, while the ordinary sexual proportion of deaths is as 109 males to 100 females. The half nearly (208 of the 420) were under 20 years of age; while the half of the deaths during the five previous years, were under three years and ten months. 21.9 per cent. of the whole deaths enumerated during the epidemic, took place under the age of 12 months; while the average at that age, during the same months of the five previous years, was 26.6 per cent. The same proportions are observed during the second year of life. These ratios in favour of early life, during the epidemic, continue until the 30th year, when it changes to the prejudice of adult life. For instance, between the years of 45 and 49, the ratio of deaths to the whole was 6.2 per cent.; while at the corresponding ages, in the five previous years, it was only 2.7 per cent. Nearly about the same disparity obtains at the quinquennial periods of 65 and 69, and through all the advanced years of life, compared to the ordinary rate of the mortality at those periods of life, during former years.

In corroboration of the great increase of mortality during the epidemic, and of the greater extent to which it bore upon those in advanced life, Dr. B. brought forward several notices and reports that have been published since the epidemic, and especially those by Drs.Clendinning,Heberden,and Graves; and regretted that reports of the kind had not been more general, and more exact in all contemporaneous conditions of the atmosphere and of the locality affected, so that some deductions might have been made, as to the laws under which the epidemic appeared and marched over the kingdom. By observing the date of its appearance, and its culminating point at many places, the nosometrical lines might be traced over the map of a country, and even over the inhabited part of the globe itself.

*Wednesday.*

The first paper read was by Dr. Macintosh, "On Dysmenorrhœa." Of this, for obvious reasons, the Athenæum gives no account, and we must defer a full report of it to a future opportunity.

Sir James Murray then read a paper, in continuation of a former one, published in the Dublin Medical Journal for July, 1836. His object was to shew, that a great variety of disordered conditions of the nerves, and of the vital organs, result from the presence of urinary secretions in the circulating fluids. In two cases of fatal neuralgia, one tic-douloureux of the thumb, he found the investments of the nerves studded with microscopic crystals. The crystals had the same constituents as those sometimes observed in the sediments of urine. Crystalline frost-work has been lately observed in the heart, brain, and other organs, by Mr. George, and in the membranes of the bowels by Professors Harrison, Apjohn, and others. Great constitutional and local irritation, he considers, is sometimes created by the acrimony of saline solutions remaining in the solids and fluids of the body; and, as these extraneous substances do not amount to tangible particles and concretions, that the presence of unsaturated alkaline or acid re-agents, maintain many obstinate disorders—as hysteria, some kinds of asthma, indigestion, &c. He had detected uric acid, urea, and many other excretory substances, in obstinate sores, in discharges from the eyes, in the incrustations of tinea capitis and lepra. He also thought the tubercles deposited in the lungs, and composed of the substance called soluble extractive, animal matter of a similar kind. When this matter meets oxygen in the lungs, it is deposited in a solid state of various consistency. The various oxides it meets with in other parts of the body, render it insoluble, and it is found imbedded in many other parts, but not so frequently as in the lungs. The elements of these extraneous matters are not always separated by the kidneys, and untoward chemical combinations are frequently set up by the presence of acid or exciting atoms not duly excreted from the fluids.

He proposed to neutralize or precipitate these impregnations by baths, acid or alkaline, as were required, and to try to obtain precipitates in the urine similar to those which pass off in the crises of fevers and acute rheumatism. Sir James concluded by lamenting, that the ridicule thrown upon such examinations, by the mystical and pretended diagnoses of charlatans, had deterred medical men from examining the secretions with sufficient precision; but hoped that the difficulty of this enquiry would no longer retard the investigations of such prominent signs and causes of disease.

Dr. James Johnson said, that the examinations recommended by Sir James Murray were becoming more and more common. He could not attribute the state of the body impregnated with the kinds of salts described in the paper, to the cause assigned by Sir James Murray. The cause, in his opinion, was rather a defect of action in the kidney, than absorption—not but that absorption and re-absorption of urea and uric acid would take place as described.

The secretary then read a paper by Dr. Madden, communicated by Professor Alison, being "Experiments on the connexion between Nerves and Muscles." The author began by stating, that two different opinions on the subject of his paper were entertained—the one by Dr. Whytt, and the school of neuralogists who attribute muscular motion to nerves and irritants, mechanical or chemical, which excite motions through nervous agency; and the other, that the power was inherent in the muscles themselves, the nerves being only conductors. He had been in consequence induced to institute a series of experiments; and he now detailed a great number on the nerves, muscles, and heart of frogs, by galvanism, mechanical irritations, by immersion, and application of narcotic solutions, and by the internal administration, or rather putting into the mouth, of prussic acid, carefully timing the progress. These were thought to bear out the following conclusion, at which the author arrived. When we see narcotics have, by no means, a destructive influence on irritability, nor on nervous trunks, no change on muscular fibre—when we see nerves cease to excite contractions *long before* muscles themselves have lost their irritability, their number and size bearing no proportion to irritability—when we see many muscles insensible to the irritation of nerves, and that a muscle with a divided nerve can recover its exhausted irritation in so short a time, and so perfectly, we must withhold our confidence in neuralogy, and believe that muscular contractility is not dependent on nervous influence.

The secretary then read a paper from Dr. O'Bryan Bellingham, "On the order of the succession of the Motions of the Heart." On performing some experiments on frogs, his attention was directed to the order of the progressive motions of the heart, which was in opposition to that given by Dr. Hope, and the one usually received, which is the following:—

| | | |
|---|---|---|
| 1st motion | the | auricular systole. |
| 2nd | — | ventricular, with impulse, and no pulse. |
| 3rd | — | ventricular diastole. |
| 4th | — | interval of ventricular repose, towards the termination of which auricular systole takes place. |

While the experiments and observations of Dr. Bellingham shewed the following:—

| | | |
|---|---|---|
| 1st was | the | auricular systole. |
| 2nd | — | ventricular diastole and impulse. |
| 3rd | — | ventricular systole. |
| 4th | — | interval of ventricular repose towards the termination — auricular systole. |

He found that, in duration of time, the diastole of the ventricles occupies double the time of the systole, and the interval of repose equalled nearly the time taken up in the systole.

With respect to the sounds, he found the

| | | |
|---|---|---|
| 1st | synchronous with | ventricular diastole. |
| 2nd | — | ventricular systole. |

Dr. Williams said these experiments had been described long ago. They were formerly supported by Dr. Corrigan, who had publicly abandoned them. They were true as to frogs, but *not* of warm-blooded animals—they were not true in the human subject, and the analogy which the author implied between the progressive motions of the heart of frogs and man was utterly impossible.

The next paper was entitled, "Observations on the disease called Cocobâe by the Africans, or Arabian Leprosy, the Ara-apatta of the Caribes of Guiana, the Radesyge of Northern Europe; and on the Methods found most effectual in the Treatment," by Dr. John Hancock. —Dr. Hancock considered that Lepra Arabum, Cocobâe of the Africans, Ara-apatta of the Caribes of Guiana, as identical, and to consist in a vitiated state of the blood and serous fluids, with obstruction of the absorbing and secerning vessels, with a peculiar diathesis, forming under the skin tubercles, knobs, and indurations, which characterize the disease. The progress being slow, the humour solidifies almost as fast as it transudes. Dr. Hancock had never known this disease to be communicated by husband to wife, or *vice versa*. This, with many other circumstances, induced him to believe that the disease was not contagious. The disease, in its early stage, is curable, though considered to be incurable. The unhappy subject is regarded with suspicion; and when the disease is fully established, the sufferer is obliged to be sent to some retired part of the colony. Bathing, salines, and anodynes, were recommended. The Indians, especially the aborigines of Guiana, resort to fomentations, baths, and a drink of the bark of a tree called Mouca, with the root of a vine, Paramaroora, a species of Cissus, and the bark of Waiacano (guiacum), the infusion of which is fermented with honey. They use, also, the bark of the tree "tamootu," a nondescript.

---

TRANSPOSITION OF THE VISCERA.

For the following interesting case we are indebted to Dr. Jamieson, of Newtownards.

Mr. Wm. J. Henry, a young gentleman of considerable talent, and a distinguished literary student of the Belfast College, enjoyed *good health* up to Feb. 1836, when symptoms of phthisis began to be manifested. Shortly after this time, on examining him with the stethoscope, I found the heart to be on the right side of the chest, and on a further examination of him by myself and other medical men, a lateral transposition of the viscera was believed to exist. The malposition of the heart, &c. was unknown to my patient and his family until I observed it. He died on the 29th of March last, ætat 19 years.

As only a few minutes were given me to perform the post-mortem examination, I had not time to look for minute departures from the normal position of parts. The following is what was observed.

*Chest.*—Right lung two-lobed and notched, and on the heart; left lung three-lobed (both studded with tubercles). Heart of the usual size, and healthy, on the right of the sternum, lying almost transversely, its apex pointed between the fourth and fifth rib, an inch external to the nipple; the pulmonary artery did not pass in front of the aorta, but to its right side, sending its left branch through the arch.

*Abdomen.*—Liver in the left hypochondrium, of the usual size, and healthy; spleen in the right hypochondrium, considerably larger than usual. Large end of the stomach to the right, pyloric orifice to the left. Cæcum attached to the left iliac fossa. The first part of the colon ascended a few inches from the cæcum, then passed through the mesentery, between the small intestines and spine, coming out below the right kidney, where it was thrown into numerous flexuses, which were attached to the right iliac fossa. The peritoneum, instead of descending as usual, after investing the stomach, passed at once into the spine; of course, neither the gastro-colic omentum nor the transverse meso-colon existed. A mass of omentum, the size of two oranges, adhered to the right iliac region. Kidneys normal.

D. JAMIESON, M.D.

*Newtownards, 22d July*, 1837.

### Sudden and Fatal Hæmoptysis.

It is not often that a hæmorrhage from the lungs is instantaneously fatal. The following is, however, an example.

A gentleman of fortune and title, aged 40 years, had been subject to cough and expectoration for seven or eight years, which seldom confined him to the house, or prevented him from enjoying the sports of the field, to which he was inordinately attached. He was also corpulent, and fond of the pleasures of the table. In the Autumn of 1836 his cough was more troublesome than usual, after an attack of influenza, and he determined to spend a Winter at Nice. He consulted Dr. Johnson, and also Dr. Chambers, that year, but, as he was of a very irritable and impatient temper, a very accurate examination of the chest could not be made. There was no idea, however, entertained by either of the above gentlemen that more than chronic bronchitis existed. He proceeded to Nice, but late in the season, and was unfortunate in the weather on the route. Between Lyons and Nice he caught cold, and began to exhibit streaks of blood in the expectoration, which was of a muco-purulent character. This tinge of the sputum never afterwards ceased. At Nice he was uncomfortable, and he travelled home much too early in the Spring of 1837, arriving in London in the month of March. His appearance was singularly altered. He had lost nearly thirty pounds weight—his pulse was above 100—the left side of the chest, especially the middle and upper portions, sounded very dull, and evinced but little respiration, and that bronchial. He had such a horror of the stethoscope, however, that a good examination of the chest could not be made. Sir H. Halford and Dr. Johnson had now the care of the patient. The expectoration was decidedly muco-purulent, tinged considerably with blood. Yet he could lie on both sides in bed, had a moderate appetite, and went backwards and forwards to his country-seat, nearly 100 miles from London. With the exception of some increase of emaciation, there was no material change in the symptoms, till the morning of Thursday, the 3d of August, when he suddenly brought up a pint or more of blood. The hæmorrhage almost immediately ceased, but strong acids were given through the day. At 7 o'clock in the evening, Dr. Chambers and Dr. Johnson saw the patient, and continued the treatment. At 8 o'clock a smaller hæmorrhage, and the patient was cupped. Friday morning another hæmorrhage of more than a pint, notwithstanding the acetate of lead and opium. Ruspini's styptic was employed in addition, and there was no more hæmorrhage till Saturday night at 11 o'clock, when the unfortunate patient sprang out of bed, discharged a considerable quantity of blood, fell on the floor, and instantly expired.

*Examination by Mr.* H. J. Johnson, *Sunday Evening, 6th August,* 1837.

The body was not emaciated to any thing like an extreme degree.

*Chest.*—The heart was a little, and but a little, larger than natural. Its right side was rather loaded with fat. Its substance was soft, and slightly lacerable.

There were about five drachms of fluid, of a yellow colour, in the pericardium.

The right lung was perfectly healthy, with the exception of a few scattered, crude, and common tubercles. These were mostly of a dark colour.

The left lung was much more diseased. Its upper lobe was universally consolidated, from grey tuberculous matter, except where it was excavated by vomicæ. One large vomica, capable of holding an egg, occupied the internal surface of this lobe, at the root of the lung, and precisely where the great bronchial tube enters it. The bronchus itself was abruptly truncated by ulceration, and its ulcerated ends, as well as its immediate vicinity, were the seat of fungous and semi-cartilaginous thickening. From the appearance of the parts, and from the situation of the vomica, it would appear that some large branches of the pulmonary artery and veins must have opened into it, and both the vomica and bronchus were filled with partially coagulated blood.

The large vomica communicated with several smaller and more recent ones, scattered through the remainder of the lobe, especially its apex.

The lower lobe was the seat of numerous tubercles, some solitary, some aggregated, but none of them yet softened.

This lung was pretty generally adherent to the side of the chest, and the adhesions were particularly strong at the apex.

